# A Data-Driven Approach to Appraisal and Selection at a Domain Data Repository

**DOI:** 10.2218/ijdc.v12i2.500

**Published:** 2017-12-31

**Authors:** Amy Pienta, Dharma Akmon, Justin Noble, Lynette Hoelter, Susan Jekielek

**Affiliations:** ICPSR, University of Michigan; ICPSR, University of Michigan; ICPSR, University of Michigan; ICPSR, University of Michigan; ICPSR, University of Michigan

## Abstract

Social scientists are producing an ever-expanding volume of data, leading to questions about appraisal and selection of content given finite resources to process data for reuse. We analyze users’ search activity in an established social science data repository to better understand demand for data and more effectively guide collection development. By applying a data-driven approach, we aim to ensure curation resources are applied to make the most valuable data findable, understandable, accessible, and usable. We analyze data from a domain repository for the social sciences that includes over 500,000 annual searches in 2014 and 2015 to better understand trends in user search behavior. Using a newly created search-to-study ratio technique, we identified gaps in the domain data repository’s holdings and leveraged this analysis to inform our collection and curation practices and policies. The evaluative technique we propose in this paper will serve as a baseline for future studies looking at trends in user demand over time at the domain data repository being studied with broader implications for other data repositories.

## Introduction

Data repositories work to ensure data are sufficiently preserved, accessible and understandable now and in the future. Yet most repositories will encounter more data than they can ingest, especially considering that the vast majority of research data are not readily usable without considerable transformation and annotation. Of critical importance to data repositories, then, is the ability to identify the most promising data for the communities they serve, so that they can apply limited resources to acquire and/or curate those data. We propose a data-driven approach to informing these collection decisions.

Demand for secondary data encapsulates both the substantive interests of the user looking for data as well as the methodological requirements for their intended data analyses (e.g. time period, sampling, geography, measures for their models, and so on) and requirements for data quality (data that are usable and documented). A scientist’s substantive needs are influenced by her theoretical and conceptual framework, prior work and its gaps, and the ever-evolving dialogue in her discipline. It also reflects the broader sociopolitical environment, including current events, that push researchers to seek new data to answer society’s pressing challenges. Repositories capture information about how various users interact with repository systems that can be used to characterize demand for data and reveal potential gaps in their holdings. In this paper, we propose several different means of measuring user demand that leverage the web analytics that most digital repositories already capture to some degree. Repositories can use our technique to assess user demand and more effectively influence collection development policies and curation activities.

## Background

We broadly define user demand for secondary data according to the attributes users employ to find data (i.e. search terms) and the attributes of the data that researchers actually download. The evaluative technique that we set forth stems from the library and information literature on user behavior in online environments. Libraries are increasingly using web analytics to better understand user behavior (Kelly, 2014; Mills, 2015). Web analytics is a type of user behavior data captured by examining the traces of information that come from human-computer interaction (Dumais et al., 2014). Online environments can be evaluated by capturing meaningful interactions with their user communities and offer a naturalistic view of human behavior versus lab studies, which offer greater experimental control but are from an artificial setting. Farney and McHale (2013) note that many libraries are using web analytics (e.g. Google Analytics) as the primary way to collect, analyze, and report data on website users and their behaviors. Also, a number of academic libraries have used Google Analytics to inform website redesign, such as the Rutgers-Newark Law Library for the Center of Law and Justice (Fang, 2007), the Morris Library at Southern Illinois University Carbondale (Arendt and Wagner, 2010), and the Health Sciences Libraries of the University of Minnesota (Loftus, 2012) and to set their content selection policy (e.g. Concordia University Libraries [Mills, 2015]). Despite the widespread use of Google Analytics among academic libraries to understand their patrons’ search needs, data archives have not typically used such information to define content demand.

Search and download behavior statistics are useful for informing an organization about unmet needs for content (Link, Tosaka, and Weng, 2015). Analysis of user behavior may inform the collection practices of the repository so that any highly searched topics where content is clearly limited can be enhanced with new content. Characteristics of users’ interactions with online resources have been used in other information science domains to make decisions about what content should be purchased, managed, or created for its user communities to make most efficient use of limited resources (Mills, 2015). Libraries, for example, have used site search metrics to build their collection development practices. Search phrases entered into a library website reveal information about content that users want to find that may be missing from the site (Fagan, 2014). One of the advantages of site search metrics is that they are much less costly than human-computer interaction studies to enhance understanding of the user experience (Hess, 2012).

In addition to site search data from Google Analytics, transaction logs and other administrative data can be used to obtain insight into user behavior at a data repository. Link, Tosaka, and Weng (2015) used multiple types of administrative usage data, such as purchasing records, circulation transactions, and interlibrary loan requests, to evaluate user needs and to inform collection development at the College of New Jersey. Others have used transaction logs to understand preferences of users (Borgman et al., 2015; Chapman et al., 2013; Dogan et al., 2009). Dogan et al. (2009) investigated PubMed users’ needs and behaviors through the analysis of log data. They found that users’ decisions were affected by the size of result sets and that Pubmed users search more persistently in comparison to general Google search users. Repositories keep download logs that can be used to describe users and use of data – especially in combination with the attributes of the data downloaded – as Borgman et al. (2015) demonstrated using log records from the Digital Archiving and Networked Services of the Netherlands (DANS). Qualitative data, such as interviews with key informants, are often used along with quantitative usage data to determine collection development strategies (Morrisey, 2010).

The analysis below is taken from the Inter-university Consortium for Political and Social Research (ICPSR), a leading domain repository for the social sciences. ICPSR has been delivering public-use data to its user communities for 55 years. Based on previous literature and the a priori understanding of common user interactions on the ICPSR website, we anticipate that analyzing user site search behavior will uncover valuable trends, identify potential gaps in holdings, and thus generate findings that could inform ICPSR’s collection development policy. While this work is largely exploratory, it was guided by several broad research questions: 1) Are there patterns in users’ search queries that can provide information about areas in which the holdings are thin? 2) What are the most popular types of user searches (e.g., keyword, study title) for social science data, and which type(s) are most helpful in identifying content gaps? and 3) How can the information about in-site searches from Google Analytics be compared to the holdings to help identify popular content areas for which there are few study matches, suggesting a potentially important gap in coverage for the repository?

## Data and Methods

### Data

We collected the data for this study using Google Analytics, focusing on search behavior captured through the search box displayed throughout ICPSR’s website, which receives over 500,000 searches each year. The search box allows visitors to search across the catalog of archived data (the largest number of searches), specific variables, and bibliographic citations, as well as ICPSR informational content. As a result, analytics of behavior using that search box capture users’ wide and varied interests. For this study, we exported search results from the Google Analytics platform from January 1, 2014 through December 31, 2014, representing 539,786 total searches. A second search analytics dataset was captured for the period of time from January 1, 2015 through December 31, 2015, representing 504,015 total searches. These two datasets encompass total unique searches, results page views/search, percentage search exits, percentage search refinements, time after search, and average search depth for each search term. As a measure of the number of users who leave the site after viewing a set of results, ‘percentage search exits’ can suggest the quality of search results (Kaushik, 2007).

### Selecting the Target Analytic Sample

We observed a long-tail distribution of the search terms visitors used on the site. For example, of 539,786 total searches in 2014, 185,445 (about 34%) were unique searches performed only a single time that year. On the other end, the most frequently used term was employed 2,727 times. In other words, a fraction of search terms were entered over and over again (a heaping on one side of the distribution where common phrases were entered hundreds, if not thousands, of times by users). A long-tail distribution means that there were also a large number of search terms that were entered only once (34%) or a couple of times. To better understand potential gaps in the repository’s holdings, we focused our analyses on the top 500 most frequently searched terms. These 500 terms represent 20.7% (111,554) of all searches in 2014, allowing us to analyze a large portion of the year’s searches in greater depth and add coding and data elements to the dataset. At the same time, the top 500 most common searches allow for considerable range in search frequency represented, with the 500^th^ most common search term employed 90 times (for a frequency range of 90 to 2,727).

### Number of Search Results Returned

Using the top 500 search terms for 2014 and 2015, we conducted our own searches of ICPSR’s holdings after the conclusion of each year’s worth of search data. The 2014 results were coded in February 2015, and the 2015 results were coded in June 2016. Staff replicated each of the top 500 searches and recorded the number of studies generated by the search results. To ensure that multi-word searches only returned the most relevant repository content, we conducted exact phrases searches by putting such searches in quotations. The repository’s search algorithm (ICPSR uses the Apache Solr search platform) looks for matches across all aspects of the study-level metadata including title, study description, study methodology, variable labels, study codebooks, and related publications. We recorded the number of matching studies both with and without quotes around the key word phrase – although we focus on the study search results generated from searches with quotes in the analysis below. The variables describing our study’s search results are an approximation of the returned search results that the user would have experienced in response to her search.

## Results

### Frequency and Volume of Site Search

ICPSR’s website offers users the ability to search for data in search boxes located on the main homepage and secondary pages and also offers additional navigation by which users can browse for data on many different types of secondary pages. Before analysing the search data described above, it is helpful to provide context around the utility of the search tools on the ICPSR website. Table 1 shows that around 50% of all ICPSR website sessions include an internal site search between 2011 and 2016. For example, in 2014, 50.03% of ICPSR website visits included an internal site search as opposed to using navigation and browsing alone^1^. These initial results also demonstrate that ICPSR website visitors perform search activities as often as coming to browse to content they need. Our subsequent analyses focus on the 539,786 searches performed in 2014 (January 1 – December 31) and 504,015 searches performed in 2015 (January 1 – December 31).

### Search Classification

We took the top 500 search terms and phrases in 2014 from the Google Analytics data and classified each into one of four mutually exclusively search types: ‘keyword or phrase’ (e.g. teen smoking), ‘named serial collection’ (e.g. Canadian National Elections and Quebec Referendum Panel Study), ‘study name’ (e.g. 1915 Iowa State Census Project), or ‘author/principal investigator name’ (e.g. John Smith). Of the top 500 searches in 2014, 73% (365) were of the ‘keyword or phrase’ type (Table 2). Just over 25% of the top searches in 2014 were for a specific study or serial collection. Only 1% referenced an author or primary investigator of a study. Searches for specific serial collections, studies, or authors may indicate a focused search for a particular study or set of studies, while keyword searches were likely less directed. We observed a similar overall pattern in the classification of 2015’s top 500 searches.

### Top Ten Keyword Searches

Based on the search behavior data, we examined the most popular keyword searches in 2014 (Table 3) – the top ten most popular keyword searches are reported in the table. Staff identified the total number of searches performed in that year that contained the popular keyword or phrase anywhere in the search. This generated an estimate of the total number of searches for any related topic. Because this includes search phrases outside the top 500, it encompasses, by association, some of the long tail of search phrases conducted infrequently during the calendar year. For example, the most popular keyword search in 2014 was ‘education.’ A total of 2,062 searches were performed on the term ‘education,’ but a total of 11,446 searches *contained* the term, including searches for ‘higher education,’ ‘educational attainment,’ ‘special education,’ ‘sex education,’ and ‘early childhood education.’

The top three keyword searches in 2014 were: education, crime, and health. ICPSR has longstanding special collections in each of these areas, and it is not surprising that many users come to the website searching for data and other content related to these topics. One of the top ten keyword search phrases in 2014 that did not reference a social science concept was ‘China.’ Interestingly, ICPSR has seen an ever-increasing volume of website traffic from China; thus China’s appearance in the top ten keyword list could be linked to the high volume of searches originating in China^2^.

We also looked at user behavior connected to the use of the top keyword searches in 2014 by examining percentage search exit, which is a measure of the portion of searches on that term that are immediately followed by the user leaving the site (Table 3). On average, 23-28% of searches on ICPSR are followed by an exit. However, searches for ‘China’ see a higher rate of 41.64%. The lowest search exit rate, 14.7%, was for searches on the keyword ‘happiness.’ Thus, data about ‘China’ may be considered higher priority for building the repository collection and/or shaping the collection development policy for the repository versus ‘happiness,’ which appears to be well-represented in the collection.

Search refinement shows how many people search again immediately following their first search (Table 4). Users searching for the top keyword terms ‘income’ and ‘race’ refined their search more than 25% of the time (27.6 and 25.7% respectively) – the highest of the top ten searches. Most of the top ten searches in 2014 were associated with session times of five or more minutes on the website (except ‘race,’ which had the lowest time after search of 4:38 minutes). The average time after search for all site searches in 2014 was 4:21 minutes. Furthermore, most of the top searches were followed by three to four page views (‘average search depth’), with the exception of ‘happiness,’ where more than eight pages on average were viewed.

The top ten search keywords and phrases in 2015 were very similar to the top keywords and phrases in 2014. As with 2014, the top three searches in 2015 were education, crime, and health (Table 4). Of the top ten 2015 search terms, nine were also in the top ten in 2014. ‘Income,’ ‘immigration,’ and ‘race’ moved up in the top ten ranking in 2015, while ‘domestic violence’ and ‘China’ moved down in the top ten ranking in 2015^3^. In 2015, the only new search term to the top ten ranking was ‘diabetes,’ which replaced ‘happiness’ as the 10^th^ most popular result.

### Identifying Gaps in ICPSR’s Collection

We wanted a method for identifying popular searches where the domain repository’s collection might fall short in meeting users’ interests. We calculated the ratio of the annual number of searches relative to the number of studies returned in the results. The premise for the development of this evaluative technique is that a high search-to-study ratio could identify potential gaps in the repository’s collection. A high number of searches on a keyword (demand), coupled with a low number of studies on that topic in the repository (depth of holdings) might indicate promising new areas for collection development. Conversely, a lower search-to-study ratio suggests that the particular topic is well-covered given the demand. Using this approach, we identified the ten keyword searches with the highest search-to-study ratios, in other words the top ten prospects for identifying gaps in the repository’s collection (Table 5).

The keywords and phrases with the highest search-to-study ratios were searched frequently (88-362 times worded exactly and 149-812 as part of a search phrase) and point to important subject areas for the data repository to consider further. The highest search-to-study ratio in 2014 was 40.6, representing 812 searches on the phrase ‘social media,’ but only 20 studies. ‘NCAA’ and ‘LGBT’ were also heavily searched yet yielded few results, pointing to possible gaps in the archive that the organization should resolve. In the case of the phrases ‘stop and frisk’ and ‘restorative justice’, it should be noted that ICPSR has a longstanding criminal justice project that promotes data on those topics to users. Thus, while a collection is being newly established at the repository it is expected that the user demand for data may be higher than the data available. Several of the ten keywords or phrases with the highest search-to-study ratio in 2014 reflected current events such as ‘stop and frisk’ and the ‘2012 election.’ The tenth highest search-to-study ratio in 2014 was 10.4 for the ‘demoralization.’ In other words, there were over ten times the number of searches as there were results returned.

In 2015, the highest search-to-study ratio was related to the search phrase ‘theatre audience’ (see Table 6). The search was conducted 108 times in 2015, but ICPSR only had four studies returned for that search phrase at that time. ICPSR’s art and culture data collection, the National Archive of Data on Arts and Culture (NADAC), was newly introduced in 2015, and the number of results was likely low as a result. However, the search phrase references the British English spelling of ‘theatre’ instead of the American English spelling of ‘theater.’ Because ICPSR has an international reach, perhaps it should consider ways to accommodate the spelling preferences of non-US audiences. Three other new search phrases had a top ten search-to-study ratio in 2015 compared to 2014. ‘Microfinancing,’ ‘sex trafficking,’ and ‘drug court’ were all frequently searched with few search results returned.

‘Social media’ remained a phrase with one of the highest search-to-study ratios. Nonetheless, the search-to-study ratio for ‘social media’ was reduced by about half from 2014 to 2015 (from 40.6 to 21.4). So, while ‘social media’ remained a highly popular search phrase in 2015 (963 searches contained the phrase), the number of search results, or studies, returned was more than doubled from 2014 (from 20 to 45). Like ‘social media,’ the phrase ‘human trafficking’ was also highly searched across both 2014 (505 searches contained the phrase) and 2015 (517 searches contained the phrase). The number of studies matching the phrase ‘human trafficking’ was still low even though the number of results returned doubled from 2014 (25 studies) to 2015 (51 studies). Human trafficking is likely related to sex trafficking as an interest area for data, suggesting the need for repositories to facilitate searches on related concepts such as these.

Taken alone, the search-to-study ratio is suggestive of gaps in the data available from ICPSR. However, these results can be used with additional information, such as quantitative and qualitative information from the broader audience of users. We turn next to our main conclusions and a discussion of how to contextualize the results.

## Conclusion

Users visit online data repositories to find data that will serve their purposes, and their interests range from the general to the specific. The ability of a data repository to fulfill its users’ needs, both now and into the future, relies on a thorough understanding of what users are searching for and how well the content matches their needs. Libraries, archives, and repositories have used web analytics to better understand user behavior on their website, but have not necessarily leveraged such information to inform collection development and ultimately better support users’ data needs (Mills, 2015). By analyzing both search behavior and the extent of repository holdings at a domain data repository, we identified popular topical areas that have noticeable gaps in the repository’s collection of available data. These areas are ripe for consideration as the repository shapes its collection development policy and allocates resources to attract high value data.

At the data repository, site search is very popular among users visiting the website. The data repository users conduct over 500,000 searches annually – mainly searching for data and related metadata. By analyzing these searches we noticed several patterns. Approximately a third of the total searches are completely unique, entered into a search box only once. We considered analyzing the single and low frequency searches in addition to the top 500 search phrases. However, among the single and infrequent searches there are many search phrases to consider, and much of the data contain misspellings and typos. Importantly, each of these search phrases tells us about the content interest of only one user (or a few users).

On the other hand, the top 500 search terms represent over 20% of the searches with the single most frequent search term telling us about the search needs of 2,062 users. Between 2014 and 2015, the top ten most popular searches were remarkably stable with nine out of ten repeating in 2015 from 2014. Thus, from a return-on-investment point of view, we recommend focusing on the most frequent searches which will account for the interests of many and could lead to strategies for effective collection development policies.

Another main finding of our analysis is that most of the searches are keyword rather than study names and/or researcher name searches. Nearly three quarters of the searches in 2014 (73%) and 2015 (69.6%) used a keyword or phrase. This finding reinforces the importance of data curation for data discovery, and perhaps enhancement of keyword/phrase curation practices. Furthermore, this finding may have implications for website and search form design that can enhance user search by keyword or phrase.

Finally, in order to identify gaps in the repository’s collection, we combined search term popularity with resulting datasets to create the search-to-study ratio. The higher the search-to-study ratio, the more popular the search phrase and/or the more limited the archives’ data holdings. Across 2014 and 2015, ‘social media’ was the search phrase associated with the highest search-to-study ratio. Social media is a relatively new area of research, and the repository has a modest number of studies that contain any information about social media. Between 2014 and 2015, the repository doubled the number of studies matching on the term ‘social media.’ Repository trends in the search-to-study ratio can be followed over time for a given phrase or keyword. The reduction of this ratio could indicate, as it appears to have for ‘social media,’ that the repository is more successfully meeting users’ needs in a particular topic area.

This study has several other important implications for data repositories that might use such strategies and techniques to guide collection development. Popular searches with a high search-to-study ratio may indicate that the repository should devote resources and effort to develop content in those areas. This might mean using resources to identify and ingest data to fill the gap and/or dedicate a higher effort to curate studies where the search-to-study ratio is high. The search-to-study ratio might also be used as an appraisal consideration when new content is offered to the repository. Given that many data repositories add and enhance metadata during curation, a list of known gaps in the collection could be used to highlight data sets and parts of data sets (such as variables) using keywords, expanding controlled vocabularies, and tagging in order to ensure the content will be returned to the user searching for content. Along with this, we identified user error such as misspellings which result in limited results, which may suggest that investing in search engines with capabilities to detect such errors would help ensure users find content.

Web analytics provide a useful, albeit limited, measure of researchers’ secondary data needs. Whether using Google Analytics data, transaction logs, or another data source for online search behavior, web analytics are strengthened when combined with other confirmatory findings. The deeper questions academic libraries and repositories are seeking to answer, such as whether users are discovering information of value to them, are best answered with a combination of methods that includes web analytics (Fagan, 2014). Data repositories should interpret results from web analytics within the broader context of some combination of the following: (1) user information – user surveys, web forms, email requests, and other modes of public feedback, (2) broader research trends – review of the research landscape including the scholarly literature, grant award databases, and expert interviews, and (3) trending topics in the news. ICPSR, for example, has used the search data presented here and the search-to-study metric matched with other information from its audience of users (e.g. feedback from ICPSR’s official representatives and generated from user surveys) and available research funding data (e.g. funding agency funding priorities).

However, we offer an additional perspective. An evaluative technique such as a search-to-study ratio has the potential to yield useful decision-making information, especially if tracked systematically and over time. User-surveys and reviews of the research landscape, when well done, take considerable resources and time. While a search-to-study ratio has limitations, it has the benefits of being simple to interpret (a ratio of 20 suggests that there are 20 searches for every one matching dataset), relatively easy to capture, and nimble for detecting and responding to patterns over time.

The search-to-study ratio is calculated based on user demand and the size of data collection, both of which may change over time. Understanding the amount of change helps to understand the value of the search-to-study evaluative technique as a tool for the data repository over time. As we saw in the case of ‘social media,’ the demand for data related to this topic stayed high between 2014 and 2015, but the size of the holdings doubled, reducing the metric by half. Beyond these individual results, overall user demand for data appears to be increasing overall along with the size of repository holdings. Between 2014 and 2015, 84 of the top 500 search phrases were new in 2015; 270 were searched more frequently; 138 were searched less frequently; and only eight were searched with the same frequency. However, over the same time period, ICPSR added 465 new studies to the repository holdings, and metadata was updated on 563 studies. As the collection of data has grown at ICPSR so have the number of search results returned. When adopting the search-to-study ratio as a collection development tool, data repositories should consider growth in user demand and the pace that their collection is growing and changing. ICPSR has a longstanding and large user base, making it an ideal case for examining such a measure and being able to use it over time. A smaller, newer repository may have a smaller audience of users making over-time comparisons more challenging.

Also, this study examined a subset of users that find data at ICPSR through an internal site search. A question for future investigation is whether findings may differ when including searches that originated from third party search engines such as Google. Dogan et al. (2009) found that PubMed users search PubMed differently (in their case, more persistently) compared to general Google search users. Many of ICPSR’s users and potential users enter the website via a Google search. As a result, our classification of the search phrase data likely undercounts searches for particular studies or serial collections. Users who start at the repository homepage (as opposed to starting via a Google search) are more likely to be exploring the collection to see what is available on a topic. They likely also know something about the repository and its collection even if they do not have a particular dataset in mind. Thus, user search data are limited to understanding the interests of a motivated and more inquisitive set of users. Data repositories also need to understand the interests and information seeking behavior of other users, such as those who enter the website directly to view a Google search result, and also potential users, who are not searching at all, but who might come to view data if the right data were available.

Finally, we also note that the number of results returned does not equate to the quality of the results returned. Many results may be returned, but this does mean that any necessarily satisfy the user’s interest. Conversely, a popular data set that covers a wide range of topics may be one of only a few results returned, but it effectively meets the needs of a large number of users. Future research could delve more deeply into this question, perhaps by combining the approach used in this paper with search refinement techniques, such as ‘time between search’ and ‘revision of queries.’ Nonetheless, we think that repositories should be aware of popular content and extend its data holdings where appropriate. Ideally, repositories successfully identify data that users will use today as well as data that future researchers will need. These are not entirely overlapping goals for understanding the utility of data that come into the repository, and predicting future use remains a significantly challenging task. However, we suggest that using an evaluative technique like the search-to-study ratio in combination with input from other sources can help domain data repositories meet such challenges.

## Figures and Tables

**Table 1.** Frequency and total volume of site search on ICPSR’s website, 2011-2016.

	Year					

	2011	2012	2013	2014	2015	2016

% Non-bounce visits that used site search	49.37	49.17	49.99	50.03	48.5	48.52
Total number of unique searches	448,350	461,639	513,824	539,786	504,015	525,876

**Table 2.** Classification of the top 500 search terms/phrases from ICPSR’s website, 2014 and 2015

	2014		2015	
	
	N	%	N	%
	
Keyword	365	73	348	69.6
Serial Collection	51	10.2	58	11.6
Study	79	15.8	90	18

PI/Author	5	1	3	0.6

**Table 3.** Top keyword searches and user behavior from Google Analytics from ICPSR’s website, 2014.

Search Phrase	# Exact Phrase Searches	% Search Exits	% Search Refinements	Average Time after Search	Average Search Depth	# Searches Containing Phrase
education	2,062	24.68%	19.06%	0:05:22	3.58	11,446
crime	1,591	23.76%	16.55%	0:05:37	3.75	14,710
health	1,156	23.62%	17.11%	0:06:04	3.93	20,777
china	1,011	41.64%	10.67%	0:06:19	3.34	4,296
income	971	19.26%	27.57%	0:05:36	3.14	5,827
domestic violence	924	28.79%	14.75%	0:06:15	3.59	2,348
immigration	833	25.57%	17.73%	0:06:42	4.12	2,231
race	801	18.85%	25.69%	0:04:38	3.15	3,816
obesity	749	28.57%	16%	0:06:10	3.59	2,215
happiness	742	14.69%	19.30%	0:07:22	8.38	1,147

**Table 4.** Top keyword searches and user behavior from Google Analytics from ICPSR’s website, 2015.

Search Phrase	# Exact Phrase Searches	% Search Exits	% Search Refinements	Average Time after Search	Average Search Depth	# Searches Containing Phrase	2014 Order
education	1,952	22.69	19.57	0:05:47	4.38	11,016	1
crime	1,609	24.30	16.79	0:05:29	4.35	12,806	2
health	1,149	24.28	17.41	0:05:49	4.48	20,398	3
income	986	20.59	25.71	0:05:16	3.61	5,609	6
immigration	904	25.44	14.99	0:05:49	3.95	2,248	8
domestic violence	896	28.01	14.54	0:05:50	3.74	2,195	4
mental health	896	21.32	17.56	0:06:13	4.69	3,505	7
race	826	20.22	29.16	0:04:24	2.86	4,137	10
china	793	39.22	10.50	0:06:07	3.89	3,555	5
diabetes	733	45.84	12.19	0:05:11	3.88	1,245	40

**Table 5.** Top ten keyword searches with highest search:study ratio from ICPSR’s website, 2014.

Search Phrase	# Exact Phrase Searches	# Searches Containing Phrase	# ICPSR Studies	Search: Study Ratio	% Search Exits^4^	% Search Exits ^5^
social media	336	812	20	40.6	26.19	26.6
NCAA	136	323	11	29.4	35.29	28.17
LGBT restorative	216	658	25	26.3	24.07	22.8
justice	114	156	7	22.3	43.86	39.1
2012 election human	118	396	18	22	17.8	15.4
trafficking second	362	505	25	20.2	30.39	30.5
generation immigrant	255	291	15	19.4	74.51	68.73
body image	147	401	31	12.9	18.37	15.96
smiddle and frisk	88	149	14	10.6	36.36	27.52

demoralization	323	323	31	10.4	96.59	95.59

**Table 6.** Top ten keyword searches with highest search:study ratio from ICPSR’s website, 2015

Search Phrase	# Exact Phrase Searches	# Searches Containing Phrase	#ICPSR Studies	Search: Study Ratio	% Search Exits^6^	% Search Exits^7^
theatre audience	108	108	4	27	100	100
LGBT	236	822	33	24.91	22.03	20.07
restorative justice	136	194	8	24.25	33.82	28.35
social media	359	963	45	21.4	22.84	23.57
2012 election	156	320	23	13.98	8.33	10
microfinance	91	151	11	13.73	35.16	30.46
human trafficking	356	517	51	10.14	32.3	30.95
sex trafficking	151	291	41	7.1	23.18	20.96
body image	88	191	31	6.16	26.14	20.94

drug court	108	378	107	3.53	34.26	27.51

## Footnotes

1 50.03% of the 1,078,925 non-bounce sessions

2 In 2014, 5.3% of ICPSR website traffic originated from China, the highest country originating traffic to the ICPSR website, after the U.S. (69.9% of traffic).

3 It is worth noting that China remains the largest non-U.S. country originating traffic to the ICPSR website in 2015. However, 42,908 sessions originated from China in 2015 down from 2014 when the number of sessions originating from China was 46,841.

4 Exact phrase

5 Containing phrase

6 Exact phrase

7 Containing phrase

## References

[R1] Arendt J, & Wagner C (2010). Beyond description: Converting web site usage statistics into concrete site improvement ideas. Journal of Web Librarianship, 4(1), 37–54. doi:10.1080/19322900903547414

[R2] Borgman CL, Scharnhorst A, van den Berg H, Van de Sompel H, Treloar A (2015). Who uses the digital data archive? An exploratory study of DANS. Proceedings of the Association for Information Science and Technology, 52(1). doi:10.1002/pra2.2015.145052010096

[R3] Chapman S, Desai S, Hagedorn K, Varnum K, Mishra S, & Piacentine J (2013). Manually classifying user search queries on an academic library web site. Journal of Web Librarianship, 7(4), 401–421. doi:10.1080/19322909.2013.842096

[R4] Dogan RI, Murray GC, Neveol A, & Lu Z (2009). Understanding PubMed user search behavior through log analysis. Database, 2009. doi:10.1093/database/bap018 PMC2797455 20157491

[R5] Dumais S, Jeffries R, Russell DM, Tang D, & Teevan J (2014). Understanding user behavior through log data and analysis In: Olson J, Kellogg W (Eds) Ways of Knowing in HCI. Springer, New York, NY

[R6] Fagan J (2014). The suitability of web analytics key performance indicators in the academic library environment. The Journal of Academic Librarianship, 40(1), 25–34. Retrieved from https://www.sciencedirect.com/science/article/pii/S0099133313000840

[R7] Fang W (2007). Using Google Analytics for improving library website content and design: A case study. Library Philosophy and Practice, 9(2), 1–17. Retrieved from https://works.bepress.com/wfang/1

[R8] Farney T, & McHale N (2013). Introducing Google Analytics for libraries. Library Technology Reports, 49(4), 5–8. Retrieved from https://journals.ala.org/index.php/ltr/article/view/4269/4882

[R9] Hess K (2012). Discovering digital library user behavior with Google Analytics. Code4lib, 17 Retrieved from http://journal.code4lib.org/articles/6942

[R10] Jansen BJ (2006). Search log analysis: What it is, what’s been done, how to do it, Library & Information Science Research, 28(3), 407–432. doi:10.1016/j.lisr.2006.06.005

[R11] Kaushik A (2007). Web analytics: An hour a day (W/Cd). John Wiley & Sons.

[R12] Kelly EJ (2014). Assessment of digitized library and archives materials: A literature review. Journal of Web Librarianship, 8(4), 384–403. doi:10.1080/19322909.2014.954740

[R13] Link FE, Tosaka Y, Weng C (2015). Mining and analyzing circulation and ILL data for informed collection development. College and Research Libraries, 76(6), 740–755. doi:10.5860/crl.76.6.740

[R14] Loftus W (2012). Demonstrating success: Web analytics and continuous improvement. Journal of Web Librarianship, 6(1), 45–55

[R15] Mills A (2015). User impact on selection, digitization, and the development of digital special collections. New Review of Academic Librarianship, 21(2), 160–169. doi:10.1080/13614533.2015.1042117

[R16] Morrisey L (2010). Data-driven decision making in electronic collection development. Journal of Library Administration, 50(3), 283–290. doi:10.1080/01930821003635010

[R17] Pienta A, Noble J, Hoelter L, Akmon D, Jekielek S (2018). Top 500 search terms used on the ICPSR website, 2014-2015. Ann Arbor, MI: Inter-university Consortium for Political and Social Research [distributor]. doi:10.3886/E101283V1

